# Elevated expression of CD30 in adult T-cell leukemia cell lines: possible role in constitutive NF-κB activation

**DOI:** 10.1186/1742-4690-2-29

**Published:** 2005-05-06

**Authors:** Masaya Higuchi, Takehiro Matsuda, Naoki Mori, Yasuaki Yamada, Ryouichi Horie, Toshiki Watanabe, Masahiko Takahashi, Masayasu Oie, Masahiro Fujii

**Affiliations:** 1 Division of Virology, Niigata University Graduate School of Medical and Dental Sciences, Niigata 951-8510, Japan; 2 Division of Molecular Virology and Oncology, Faculty of Medicine, University of the Ryukyus, Nishihara, Okinawa 903-0215, Japan; 3 Department of Laboratory Medicine, Nagasaki University Graduate School of Biomedical Sciences, Nagasaki 825-8501, Japan; 4 Fourth Department of Internal Medicine, Faculty of Medicine, Kitasato University, Sagamihara, Kanagawa 228-8555, Japan; 5 Laboratory of Tumor Cell Biology, Department of Medical Genome Sciences, Graduate School of Frontier Sciences, The University of Tokyo, Minato-ku, Tokyo 108-109, Japan

## Abstract

**Background:**

Human T-cell leukemia virus type 1 (HTLV-1) is associated with the development of adult T-cell leukemia (ATL). HTLV-1 encoded Tax1 oncoprotein activates the transcription of genes involved in cell growth and anti-apoptosis through the NF-κB pathway, and is thought to play a critical role in the pathogenesis of ATL. While Tax1 expression is usually lost or minimal in ATL cells, these cells still show high constitutive NF-κB activity, indicating that genetic or epigenetic changes in ATL cells induce activation independent of Tax1. The aim of this study was to identify the molecules responsible for the constitutive activation of NF-κB in ATL cells using a retroviral functional cloning strategy.

**Results:**

Using enhanced green fluorescent protein (EGFP) expression and blasticidin-resistance as selection markers, several retroviral cDNA clones exhibiting constitutive NF-κB activity in Rat-1 cells, including full-length CD30, were obtained from an ATL cell line. Exogenous stable expression of CD30 in Rat-1 cells constitutively activated NF-κB. Elevated expression of CD30 was identified in all ATL lines examined, and primary ATL cells from a small number of patients (8 out of 66 cases).

**Conclusion:**

Elevated CD30 expression is considered one of the causes of constitutive NF-κB activation in ATL cells, and may be involved in ATL development.

## Background

Adult T-cell leukemia (ATL) is an extremely aggressive human CD4+ T-cell leukemia (reviewed in [1]). ATL is resistant to chemotherapy and most patients die within one year of diagnosis. Human T-cell leukemia virus type 1 (HTLV-1) infection of CD4+ T-cells is the first step in ATL development. However, this alone is not sufficient for the development of leukemia because a minority of HTLV-1 infected subjects (approximately 5%) develop ATL on average 60–70 years after the infection (reviewed in [2,3]). *In vitro*, HTLV-1 transforms primary human CD4+ T-cells in an interleukin (IL)-2-dependent or an IL-2-independent manner. HTLV-1 encoded Tax1 protein is thought to play a critical role in T-cell transformation and leukemogenesis, as Tax1 itself immortalizes primary human CD4+ T-cells *in vitro *[4,5] and inhibits apoptosis induced by various stimuli in T-cell lines [6-9].

Tax1 is a multifunctional protein (reviewed in [2,3]). It activates the transcription of many cellular genes associated with cell growth, such as genes encoding cytokines [10-13], cytokine receptors [14-17], anti-apoptotic protein [8,18], cell cycle regulators [19-22], and proto-oncogenes [23]. Those proteins are thought to contribute to the deregulated proliferation of HTLV-1-infected cells. Accumulating evidence suggests that activation of cellular genes by Tax1, particularly through the nuclear factor-kappaB (NF-κB) pathway, is a critical process in transformation as well as the inhibition of apoptosis. For example, the transforming activity of Tax1 is abrogated by mutations that impair the ability of Tax1 to activate NF-κB [24-26]. Tax1 inhibits apoptosis of mouse T-cell lines by induction of the anti-apoptotic gene Bcl-xL through NF-κB activation [8,18].

In resting T-cells, NF-κB factors are sequestered in the cytoplasm, tightly associated with inhibitory proteins IκBs. Activation of NF-κB generally involves phosphorylation and degradation of IκBs, followed by nuclear translocation of NF-κB dimers and subsequent activation of the genes containing NF-κB binding sites (reviewed in [27]). Alternatively, NF-κB activation occurs by inducible processing of NFKB2/p100 with IκB-like inhibitory activity, into p52 with DNA binding activity, followed by nuclear translocation of p52 containing NF-κB dimers (reviewed in [28]). These two processes are largely dependent on an IκB kinase (IKK) complex comprised of two catalytic subunits, IKKα and IKKβ and a regulatory subunit IKKγ/NEMO. Tax1 interacts with the IKK complex through these three subunits and stimulates the catalytic activity [29-32].

In primary ATL cells as well as cell lines established from ATL patients, NF-κB is constitutively active as seen in HTLV-1 transformed cells [33]. It appears that this constitutive NF-κB activation contributes to the survival and chemotherapy resistance of ATL cells, since treatment of ATL cells with a NF-κB inhibitor, Bay 11-7082, induces apoptosis of these cells [34]. However, how NF-κB is constitutively activated in ATL cells is still largely unknown since the tax gene is mutated in some ATL cases [35,36] or the level of expression of Tax1 in these cells is extremely low, thereby being clearly insufficient to activate NF-κB [37,38]. There may be genetic or epigenetic changes that lead to tax-independent NF-κB activation, such as a gain of function of the NF-κB activating molecule(s) or a loss of function of the NF-κB regulator(s). The elucidation of the molecular mechanism of NF-κB activation in ATL cells is quite important in the light of prevention, diagnosis and treatment of ATL.

In order to identify the molecule(s) responsible for the constitutive NF-κB activation in ATL, we took a functional screening approach using a retroviral cDNA library from an ATL cell line and a reporter cell line that is easily distinguishable as a positive clone once NF-κB is activated. We obtained several cDNA clones that constitutively activate NF-κB. One of these, the full-length CD30, is a member of the TNF receptor superfamily and a marker of malignant Hodgkin and Reed-Sternberg (H-RS) cells in Hodgkin's lymphoma (HL) (reviewed in [39,40]). It is suggested that overexpression of CD30 in H-RS cells and HL cell lines contributes to CD30 ligand-independent constitutive NF-κB activation in these cells [41]. The results showed that CD30 is strongly expressed in all ATL cell lines examined, and that CD30 is expressed in primary ATL cells in a small number of ATL patients.

## Results and Discussion

### Screening of NF-κB activating molecules

**Figure 1:**
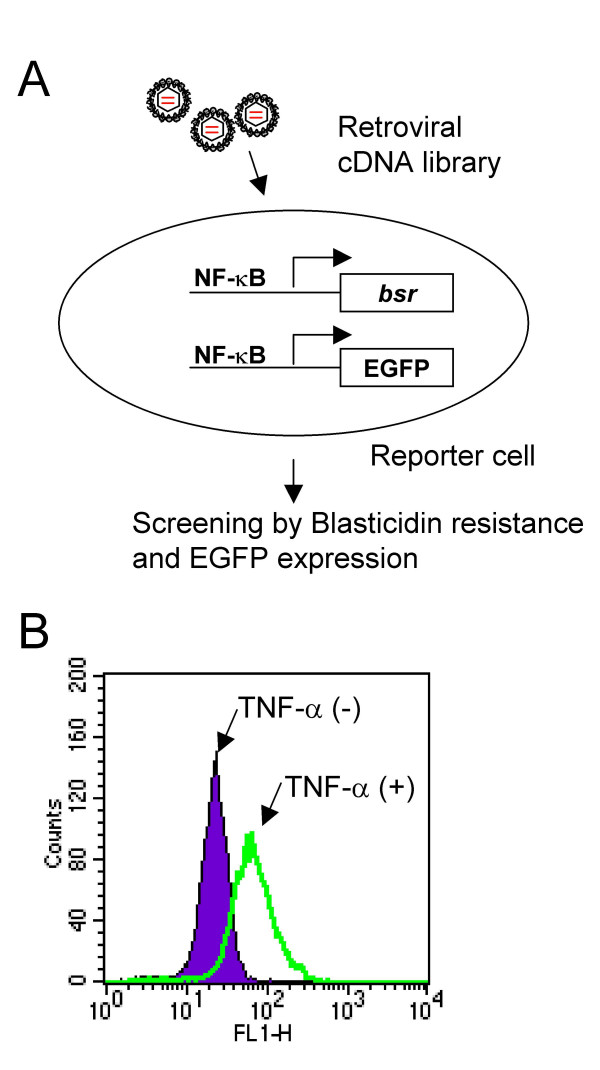
**Strategy for cloning NF-κB activating molecules**. A) A retroviral cDNA library from an ATL cell line is transduced to a reporter cell line expressing EGFP and *bsr *in response to NF-κB activation. Blasticidin-resistant and EGFP expressing cell clones are expanded and cDNA clones are obtained by PCR using the retrovirus vector specific primers. B) Visualization of NF-κB activation in reporter cells. Reporter cells were stimulated with TNF-α for 48 hours and tested for the expression of EGFP by FACS analysis.

In order to identify the molecule(s) responsible for the constitutive NF-κB activation in ATL cells, we employed a functional screening strategy using a retroviral cDNA library from an ATL cell line. In theory, if ATL cells express NF-κB activating molecules leading to the constitutive activation, it would be possible to obtain such clones using NF-κB activation as a positive selection marker (Figure 1A). We generated a retroviral cDNA library from ATL cell line TL-OmI, which had already been shown to have constitutive NF-κB activity in the absence of Tax1 [33]. As a reporter cell line, we generated a Rat-1 fibroblast cell line with a stably integrated blasticidin deaminase gene (*bsr*) fused to enhanced green fluorescent protein (EGFP) under five repeats of the NF-κB binding sequences from the IL-2 receptor α chain and the minimal HTLV-1 promoter [42]. The *bsr *and EGFP enabled us to easily identify NF-κB activated cells as surviving cells with green fluorescence in the presence of blasticidin. A pilot experiment, however, showed that the green fluorescent signal from this fusion protein in the cells after NF-κB activation stimuli (such as TNF-α treatment) was extremely low, probably due to the short half life of the fusion protein or a conformational change that interferes with EGFP activity (data not shown). Thus, we further stably transfected the EGFP gene regulated under the same NF-κB responsive promoter into the reporter cell line. This new reporter cell line, named Rat-1 κB-*bsr*EGFPx2, showed bright EGFP signals and blasticidin resistance after TNF-α treatment (Figure 1B and data not shown). This doubly transfected cell line has a critical advantage in this screening system. It is possible that retroviral cDNA is inserted near the *bsr*EGFP gene and the retroviral long terminal repeat (LTR) constitutively activates the expression of the *bsr*EGFP gene, resulting in a false positive clone. However, if it occurs in the new reporter cell line, these cells should have minimal EGFP signals because of the extremely low fluorescence intensity of the fusion protein and such cells could be easily eliminated during the screening process.

After converting the plasmid library to the retroviral library by introduction into packaging cells, the resultant viruses were transduced into the Rat-1 κB-*bsr*EGFPx2 reporter cells. After selection in the presence of blasticidin, under an inverted fluorescence microscope, EGFP-positive cells were picked up and expanded, followed by genomic DNA extraction. PCR products amplified by the primers specific for the retroviral vector were cloned and the sequences were determined. Following three independent screenings, we obtained a total of 64 clones (Table 1).

**Table 1:** NF-κB activators isolated from the TL-OmI cDNA library.

cDNA	No. of isolates	Characteristics
NIK	58	N terminal deletion
CD30	3	Full length
LT-βR	2	Cytoplasmic region
RIP2	1	Full length

NF-κB inducing kinase (NIK) is a mitogen-activated protein kinase kinase kinase (MAP3K), which is involved in NFKB2/p100 processing and nuclear translocation of p52/RelB dimers, the so-called noncanonical pathway [43]. This pathway is activated by lymphotoxin-β (LT-β), CD40 ligand, and B cell activating factor (BAFF) and depends on IKKβ (reviewed in [28]). All the NIK clones we obtained possessed the intact kinase domain and the N-terminal amino acid deletion, starting at codon 417. It has been reported that the N-terminus of NIK contains a negative-regulatory domain and an N-terminal truncation mutant has higher NF-κB inducing activity than the wild type [44]. It is likely that this deletion was introduced by incomplete reverse transcription with oligo dT primer during the cDNA library construction process. It is interesting to note that none of the other MAP3Ks that can activate NF-κB, such as MEKK1 [45], were cloned. This selective isolation of NIK as well as its high frequency among the NF-κB-inducing clones indicates that NIK and/or the noncanonical pathway may play a central role in the constitutive NF-κB activation seen in various tumors.

The sequences of the two LT-β receptor (LT-βR) clones were identical and encoded a part of the cytoplasmic domain of the receptor (from codon 268 to 395). The retrovirus vector used in our experiments transcribes two mRNAs, one spliced and one unspliced. The unspliced mRNA may translate fusion genes of *gag *with inserted cDNA when they are in frame. The isolated LT-βR clone is in frame with *gag *and could be expressed as a fusion protein, which might induce constitutive NF-κB activation. This cloned LT-βR mutant is likely to be an artificial one generated during the library construction process as discussed above.

The receptor-interacting protein 2 (Rip2) is a serine/threonine kinase that contains a caspase-recruitment domain (CARD) at its carboxyl terminus and has been shown to induce NF-κB activation in an over-expression system [46]. Rip2 has been implicated in regulating both the innate and adaptive immune responses [47,48]. Recently, it has been reported that Rip2 participates in Bcl10-mediated NF-κB activation [49]. The Rip2 clone isolated in our study is full length and not in frame with *gag*. It is possible that Rip2 is over-expressed in ATL cells and this contributes to constitutive NF-κB activation. This hypothesis is currently under investigation.

### Exogenous stable expression of CD30 induces constitutive NF-κB activation

**Figure 2:**
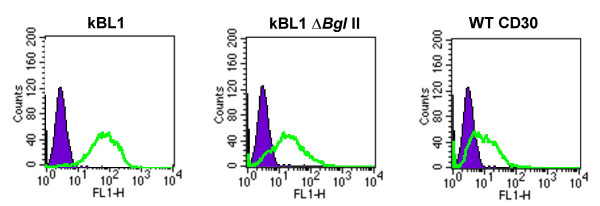
**Exogenous stable expression of CD30 induces constitutive NF-κB activation in Rat-1 cells **Rat-1 κB-*bsr*EGFPx2 cells were infected with the pMX kBL1, pMX kBL1Δ*Bgl*II, or pMX CD30WT virus and tested for the expression of EGFP by FACS analysis. The cells infected with pMX virus were used as a negative control. CD30 expression was seen in cells infected with all three viruses containing CD30 gene (pMX kBL1, pMX kBL1Δ*Bgl*II, pMX CD30WT) but not pMX virus (data not shown).

CD30 is a member of the TNF receptor super family and is known as a marker of malignant Hodgkin and Reed-Sternberg (H-RS) cells in Hodgkin's lymphoma (HL). It has been suggested that overexpression of CD30 in H-RS cells and HL cell lines contributes to CD30 ligand (CD30L)-independent constitutive NF-κB activation in these cells [41]. The same possibility in ATL cells was further examined. One of the three CD30 clones (named kBL1) contains full-length CD30 in frame with *gag *(the other two clones were not completely sequenced). As described above, the retrovirus vector used in our experiments transcribes two mRNAs, one is a spliced one and the other is an unspliced one. The unspliced mRNA can translate fusion genes of *gag *with inserted cDNA when they are in frame. To determine that the fusion between CD30 and *gag *is responsible for its constitutive NF-κB inducing activity, we generated a retroviral vector that expresses only full-length CD30 by introducing a frame shift mutation upstream of the CD30 open reading frame of the cloned gene. We also constructed a retroviral vector for full-length CD30 cDNA (pMX CD30WT) out of frame with *gag*. Retroviral vectors for CD30 either in or out of frame with *gag *(pMX kBL1 or pMX kBL1Δ*Bgl*II respectively) and pMX CD30WT were introduced into packaging cells and the Rat-1-*bsr*EGFPx2 cells were infected with the resultant viruses. After 48 hours, EGFP signals were examined by fluorescence activated cell sorter (FACS) analysis. In all three cases, CD30 induced constitutive NF-κB activation, although CD30 in frame with *gag *had stronger NF-κB inducing activity, which means the fusion with *gag *indeed augments the activity (Figure 2). This result demonstrates that stably overexpressed CD30 can induce constitutive NF-κB activation in a ligand independent manner in Rat-1 cells, as described previously in human embryonic kidney cell line 293 [41].

### Overexpression of CD30 in ATL cell lines

**Figure 3:**
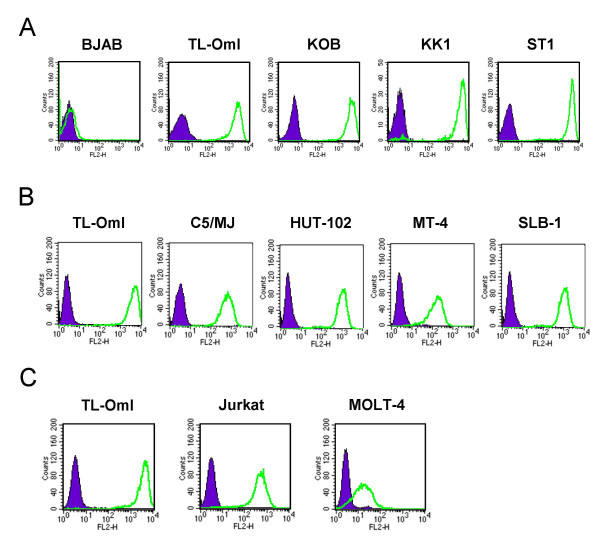
**Elevated expression of CD30 in ATL cell lines**. CD30 expression was examined in A) ATL, B) HTLV-1-transformed, and C) HTLV-1-negative cell lines by FACS analysis. A Burkitt lymphoma cell line (BJAB) was used as a negative control in A). TL-OmI was used as a standard for the CD30 expression level in B) and C).

We next examined CD30 expression in ATL-derived T-cell lines, HTLV-1 transformed cell lines and HTLV-1 negative T-cell lines using FACS analysis (Figure 3). All ATL cell lines (TL-OmI, KOB, KK1 and ST1) showed strong CD30 expression whereas a B lymphoma cell line (BJAB) showed no staining (Figure 3A). HTLV-1 transformed cell lines (HUT-102, C5/MJ, MT-4 and SLB-1) also showed CD30 expression but the amount of the expression was various and lower than TL-OmI (Figure 3B). In HTLV-1 negative T-cell lines (Jurkat and MOLT-4), the expression of CD30 was significantly lower than TL-OmI (Figure 3C). Interestingly, NF-κB activity was much lower in Jurkat and MOLT-4 than ATL cell lines. Thus CD30 expression level is well correlated with the NF-κB activity, which suggests that overexpression of CD30 might be at least one of the factors that contributes to constitutive NF-κB activation in ATL cell lines. In HTLV-1 transformed cells, NF-κB activation is thought to be largely dependent on Tax1, however it is possible that relatively strong CD30 expression in HUT-102 and SLB-1 also contributes to constitutive NF-κB activation in these cells. In addition, CD30L expressed in ATL cell lines may possibly contribute to CD30 activation by a cell-cell contact mechanism. RT-PCR analysis for CD30 ligand showed that CD30L expression in TL-OmI cells was extremely weak compared with a Burkitt lymphoma cell line (EB-1), in which CD30L is weakly expressed (data not shown) [50]. This finding suggests that CD30L is not involved in the constitutive NF-κB activation in TL-OmI cells.

### Expression of CD30 in primary ATL cells

**Figure 4:**
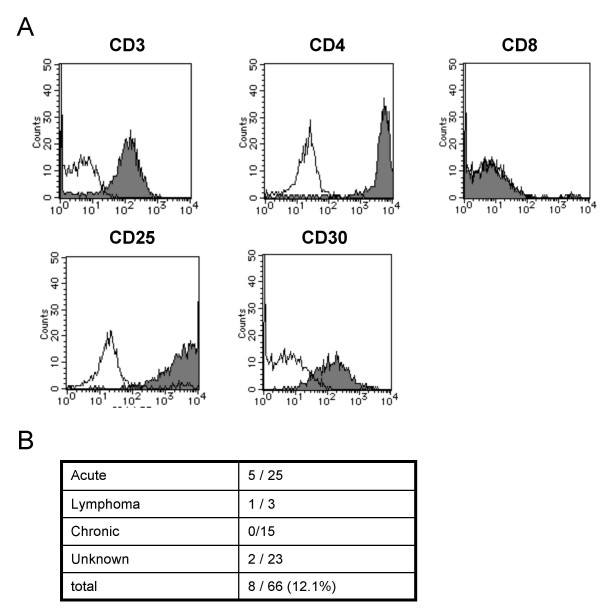
**CD30 expression in primary ATL cells**. A) Primary ATL cells from a patient (case 8) were tested for the expression of CD3, CD4, CD8, CD25 and CD30 by FACS analysis. B) Summary of the number of CD30-positive ATL cases.

Next, we examined CD30 expression in primary ATL cells by FACS analysis. Peripheral blood lymphocytes (PBLs), lymph node cells, or ascitic fluid cells from ATL patients were stained with anti-CD30 antibody (Figure 4 and Table 2). ATL cases in which more than 30% of the cells expressed CD30 were classified as CD30-positive ones. CD30 expression was seen in 8 of 66 ATL cases (12.1%) and the CD30 expression was predominantly seen in the acute type (5 of 25 cases), representing the advanced stage of ATL (Figure 4B). Data of the FACS analysis (CD3, CD4, CD8, CD25, and CD30 expression) of the CD30-positive ATL cases are summarized in Table 2.

**Table 2:** Cell surface markers in CD30-positive ATL cases

				% of Positive Cells				
				
Case	Sex	Type	Material	CD3	CD4	CD8	CD25	CD30
1	M	Acute	PB	90.1	86.5	4.4	89.8	56.5
2	F	Acute	PB	18.9	78.3	3.0	81.0	48.7
3	F	Acute	PB	94.8	14.2	64.2	81.9	84.8
4	F	Acute	PB	89.3	96.3	2.6	93.3	35.5
5	M	Acute	LN	10.1	96.6	5.2	90.1	93.0
6	M	Lymphoma	LN	8.7	85.1	5.3	58.1	76.5
7	F	Unknown	LN	67.5	77.8	23.0	81.7	60.4
8	F	Unknown	Ascites	89.5	99.7	0.1	99.5	96.2

The percentage of positive cells was determined by immunofluorescence staining with respective antibodies and flow cytometric analysis.

Abbreviations: PB, peripheral blood; LN, lymph node.

**Figure 5:**
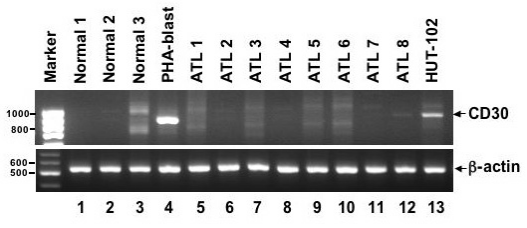
**CD30 mRNA expression in primary ATL cells **Primary ATL cells from ATL patients (lanes 5–12) and normal PBLs from healthy adult donors (lanes 1–3) were tested for CD30 (upper panel) and β-actin (lower panel) mRNA expression by RT-PCR analysis. The CD30 expression was seen in ATL8 (lane 12). PHA-stimulated PBLs (lane 4) and HUT-102 (lane 13) were used as a positive control.

It has been reported that proteolytic cleavage of membrane-anchored CD30 releases a soluble fragment corresponding to the extracellular domain [51]. To examine the possibility that the low frequency of CD30 expression in primary ATL cells in the FACS analysis is due to this proteolytic processing, CD30 mRNA expression was examined in 8 ATL cases different from those used in the FACS analysis. Strong CD30 mRNA expression was seen in HUT-102 and PBLs activated by phytohemagglutinin (PHA), whereas the CD30 expression was seen in only one case (ATL8) diagnosed as the lymphoma type (Figure 5). The amount of CD30 mRNA expression in this case was lower than HUT-102 and it might not be sufficient to induce NF-κB activation by itself. However it is possible that weak CD30 expression still contributes to the constitutive NF-κB activation in cooperation with other signaling molecules *in vivo*. In summery, these FACS and RT-PCR data suggest that the expression of CD30 in ATL is not a common event and is limited to a small number of ATL cases. This is consistent with a previous report that CD30 expression was seen in 7 out of 36 cases (19.4%) when their lymph node biopsies were immunohistochemically stained with anti-CD30 antibody [52].

The reason for the discrepancy between ATL cell lines and primary ATL cells in terms of CD30 expression is unknown at present. One possibility is that only CD30-positive primary ATL cells could be established as a cell line *in vitro *because of their stronger NF-κB activity or activation of other signaling pathways originating from CD30. In fact, CD30 activates not only NF-κB but also the mitogen activated protein kinase (MAPK) pathways, such as extracellular regulated kinase (ERK), Jun N-terminal kinase (JNK), and p38 MAPK pathways [53,54].

Recently, it has been reported that the noncanonical pathway is involved in constitutive NF-κB activation in ATL cells [55]. Although activation of the noncanonical pathway by CD30 has not yet been reported, it is likely that CD30 activates this pathway through association with TNF receptor associated factors (TRAFs) like LT-βR and CD40. In H-RS cells, which strongly express CD30, TRAF2 and TRAF5 make aggregates in the cytoplasm and co-localize with downstream signaling molecules, such as IKKα and IKKβ [56]. It would be interesting to see whether TRAF2 and TRAF5 also form aggregates in ATL cell lines and primary ATL cells expressing CD30.

In order to confirm that CD30 is involved in constitutive NF-κB activation and cell survival in ATL cell lines, we tried to knockdown CD30 expression in these cells by using short-hairpin RNAs. We generated 11 different short-hairpin RNAs for CD30 in total, but none of them showed any RNA interference effect. We also tried to introduce a decoy CD30 that lacks most of the cytoplasmic region and has been shown to induce apoptosis in H-RS cells [41], by using an adenovirus vector. However we were unable to obtain a sufficiently high titer adenovirus as a decoy CD30 mutant to carry out the experiment. Thus, whether elevated expression of CD30 actually contributes to constitutive NF-κB activation in ATL cell lines still remains unknown.

In this regard, the mechanism by which NF-κB is constitutively activated in ATL cells still remains a mystery. However, our data suggest that the elevated expression of CD30 plays a critical role in NF-κB activation in ATL cell lines and a small number of primary ATL cells. Other molecules belonging to the TNF receptor family, such as LT-βR, OX40, or downstream signaling molecules, could be involved in constitutive NF-κB activation in CD30-negative ATL cells, and the identification of such molecules would contribute to the prevention, diagnosis and treatment of ATL.

## Conclusion

ATL cells have constitutive NF-κB activity which is important for the cells' survival. This NF-κB activation is independent of Tax protein expression. By screening a retroviral cDNA library from an ATL cell line to identify NF-κB activating molecules, we obtained several cDNA clones including full-length CD30. CD30 is strongly expressed in ATL cell lines and primary ATL cells from a small number of patients. Our results suggest that elevated expression of CD30 is one of the factors responsible for constitutive NF-κB activation in ATL cells.

## Methods

### Cell culture

Rat-1, a rat fibroblast cell line, was cultured in Dulbecco's modified Eagle's medium (DMEM) supplemented with 10% fetal bovine serum (FBS). Human T-cell lines used in the present experiments have been characterized previously [33,57]. Jurkat and MOLT-4 are HTLV-1 negative human T-cell lines. HUT-102, C5/MJ, MT-4 and SLB-1 are HTLV-1-positive human T-cell lines. TL-OmI, KK1 [58], KOB [59], and ST1 [60] are HTLV-1-positive, ATL-derived cell lines. These cells were cultured in RPMI 10% FBS. Recombinant human IL-2 (Takeda Chemical Industries, Osaka, Japan) was added at 0.5 nM to the culture of KK1, KOB and ST1. A retrovirus packaging cell line Plat-E [61] was cultured in DMEM 10% FBS containing 1 μg/ml puromycin (Calbiochem, La Jolla, CA) and 10 μg/ml blasticidin (Invitrogen, San Diego, CA).

### cDNA library construction

Poly (A)^+ ^RNA was purified from TL-OmI using FastTrack 2.0 (Invitrogen). cDNA was synthesized by oligo(dT) primers using SuperScript Choice System (Invitrogen) according to the instructions provided by the manufacturer. The resulting cDNAs were size-fractionated through agarose gel electrophoresis, and cDNA fragments longer than 2.5 kb were extracted from the gel by using Qiaex II (Qiagen, Hilden, Germany). The cDNA fragments were then inserted into *Bst*XI sites of the retroviral vector pMX [62] using *Bst*XI adapters (Invitrogen). The ligated DNA was ethanol-precipitated and then electroporated into DH10B competent cells (Electromax DH10B; Invitrogen). About 1 × 10^6 ^independent clones were amplified on 150 mm LB/amp plates and plasmid DNA was purified by using Qiagen Plasmid Giga kit (Qiagen).

### Generation of a reporter cell line

The NF-κB reporter plasmid κB-EGFP was constructed by replacing the luciferase gene (a *Bgl*II – *Bam*HI fragment) of the κB-Luc plasmid [42] with EGFP (a *Hind*III – *Afl*II fragment) from pEGFP-N3 (Clontech Laboratories, Palo Alto, CA) by blunt-end ligation. To construct the plasmid κB-*bsr*EGFP, which expresses *bsr*EGFP fusion protein, a PCR amplified *bsr *gene fragment was inserted in the *Apa*I and *Bam*HI sites upstream of EGFP of the κB-EGFP plasmid. To prepare a NF-κB reporter cell line, Rat-1 cells (5 × 10^6^) were transfected with 20 μg of κB-*bsr*EGFP and 1 μg of pcDNA3 (Invitrogen) by electroporation at 250 V and 975 μF. The transfected cells were cultured in 500 μg/ml G418 (Invitrogen), and resistant clones were screened for EGFP signals after being infected with retroviruses that express Epstein-Barr virus transforming protein LMP1. The selected cell clone (Rat-1 κB-*bsr*EGFP) was further transfected with κB-EGFP and pMik-HygB and cultured in 250 μg/ml hygromycin B (Wako Pure Chemical Industries, Osaka, Japan). Resistant clones were screened for EGFP signals after stimulation with 20 ng/ml TNF-α (Peprotech, London, UK).

### Preparation of retroviruses and infection of reporter cells

Plat-E cells (2 × 10^6 ^cells) were seeded onto 60 mm dishes one day before transfection. The cDNA library (3 μg) was transfected using Fugene 6 (Roche Molecular Systems, Inc., NJ) according to the protocol provided by the manufacturer. Cells were cultured for 48 hours and the retroviral supernatant was harvested. For infection of reporter cells, 2.5 × 10^5 ^cells were seeded onto 100 mm dishes one day before infection and incubated with 10 ml DMEM 10% FBS containing 0.6 ml of the virus stock for 24 hours in the presence of polybrene (20 μg/ml). The medium was changed to fresh DMEM 10% FBS after 24 hours. After another 24 hours, the cells were incubated with medium containing 50 μg/ml blasticidin (Invitrogen).

### Isolation of cDNA fragments from blasticidin-resistant clones

Genomic DNA was extracted from the blasticidin-resistant clones by DNeasy kit (Qiagen) and subjected to PCR to recover integrated cDNAs using pMX vector primers (5'-GGTGGACCATCCTCTAGACT-3' and 5'-CCCCTTTTTCTGGAGACTAAAT-3'). The PCR products were cloned into pGEM-T Easy vector (Promega, Madison, WI) and sequenced using BigDye Terminator v1.1 cycle sequencing kit (Applied Biosystems, Foster City, CA).

### Expression plasmids

The retroviral vector pMX LMP1 was prepared by inserting an *Eco*RI – *Bam*HI fragment of pSG5 F-LMP1 [63] in the *Eco*RI site of pMX by blunt-end ligation. pMX CD30WT was generated by inserting a *Mlu*I – *Not*I fragment of pCD30WT [64] in the *Eco*RI and *Not*I sites of pMX by blunt-end ligation. pMX kBL1 was generated by inserting a *Bam*HI – *Sfi*I fragment of pGEMT kBL1 in the *Bam*HI – *Not*I sites of pMX. The *Bgl*II site of pMX kBL1 was destroyed by cutting by *Bgl*II, filling in by T4 polymerase, and self-ligation to make pMX kBL1Δ*Bgl*II.

### Flow cytometric analysis

Heparinized peripheral blood, a piece of a lymph node, or ascites (in case no. 8) was collected from patients with ATL after obtaining informed consent in accordance with the Helsinki Declaration. Mononuclear cells were separated by Lymphoprep™ density gradient centrifugation (Axis-Shield PoC AS, Oslo, Norway). Morphological and surface marker analyses indicated that ATL cells in these samples always accounted for more than 80% of the total cell population in most cases. The study protocol was approved by the Human Ethics Review Committee of Nagasaki University Graduate School of Biomedical Sciences.

Primary ATL cells or T-cell lines were incubated for 30 min at 4°C with each PE-labeled or FITC-labeled monoclonal antibody (mAb). Cells were also incubated with isotype matched control antibodies. The following antibodies were used: PE-labeled mouse anti-human CD4 and CD25, FITC-labeled anti CD3 and CD8 (BD Biosciences Pharmingen, San Diego, CA); and PE-labeled mouse anti-human CD30 (Dako Corporation, Carpinteria, CA or Immunotech, Marseille, France). After washing with PBS, the cells were analyzed on FACScan flow cytometer using Cellquest software (Becton Dickinson, San Jose, CA).

### Reverse transcription-polymerase chain reaction

Total cellular RNA was extracted with Trizol (Invitrogen) according to the protocol provided by the manufacturer. First-strand cDNA was synthesized from 1 μg total cellular RNA in a 20-μl reaction volume using an RNA PCR kit (Takara Shuzo, Kyoto, Japan) with random primers. Thereafter, cDNA was amplified for 35 cycles for CD30 and 28 cycles for β-actin. The oligonucleotide primers used were as follows: for CD30, sense, 5'-CTGTGTCCCCTACCCAATCT-3' and antisense, 5'-CTTCTTTCCCTTCCTCTTCCA-3'; [65] and for β-actin, sense, 5'-GTGGGGCGCCCCAGGCACCA-3' and antisense, 5'-CTCCTTAATGTCACGCACGATTTC-3'. Product sizes were 860-bp for CD30 and 548-bp for β-actin. Cycling conditions were as follows: denaturing at 94°C for 45 sec (for CD30) or for 30 sec (for β-actin), annealing at 62°C for 45 sec (for CD30) or 60°C for 30 sec (for β-actin) and extension at 72°C for 60 sec (for CD30) or for 90 sec (for β-actin). The PCR products were fractionated on 2% agarose gels and visualized by ethidium bromide staining.

## Competing interests

The author(s) declare that they have no competing interests.

## Authors' contributions

MH carried out the cDNA cloning and the functional analysis of CD30. TM and NM carried out the RT-PCR analysis. YY carried out the FACS analysis. MH, RH, TW, MT, MO and MF participated in the experimental design, data interpretation, and writing of the manuscript.
